# Vascular smooth muscle cells remodel collagen matrices by long-distance action and anisotropic interaction

**DOI:** 10.1007/s11517-012-0916-6

**Published:** 2012-06-07

**Authors:** Jeroen van den Akker, Bilge Guvenc Tuna, Adrian Pistea, Arie J. J. Sleutel, Erik N. T. P. Bakker, Ed van Bavel

**Affiliations:** Department of Biomedical Engineering and Physics, Academic Medical Center L0-120, University of Amsterdam, PO Box 22660, 1100 Amsterdam, The Netherlands

**Keywords:** Collagen, Smooth muscle cell, Transglutaminase, Biomechanics, Remodeling

## Abstract

While matrix remodeling plays a key role in vascular physiology and pathology, the underlying mechanisms have remained incompletely understood. We studied the remodeling of collagen matrices by individual vascular smooth muscle cells (SMCs), clusters and monolayers. In addition, we focused on the contribution of transglutaminase 2 (TG2), which plays an important role in the remodeling of small arteries. Single SMCs displaced fibers in collagen matrices at distances up to at least 300 μm in the course of 8–12 h. This process involved both ‘hauling up’ of matrix by the cells and local matrix compaction at a distance from the cells, up to 200 μm. This exceeded the distance over which cellular protrusions were active, implicating the involvement of secreted enzymes such as TG2. SMC isolated from TG2 KO mice still showed compaction, with changed dynamics and relaxation. The TG active site inhibitor L682777 blocked local compaction by wild type cells, strongly reducing the displacement of matrix towards the cells. At increasing cell density, cells cooperated to establish compaction. In a ring-shaped collagen matrix, this resulted in preferential displacement in the radial direction, perpendicular to the cellular long axis. This process was unaffected by inhibition of TG2 cross-linking. These results show that SMCs are capable of matrix remodeling by prolonged, gradual compaction along their short axis. This process could add to the 3D organization and remodeling of blood vessels based on the orientation and contraction of SMCs.

## Introduction

Arterial structure is normally well matched to the functional needs. Thus, presence and organization of elastin and collagen fibers ensures the non-linear stress–strain relation required for a stable diameter under pressure, and matrix strength is sufficient to withstand pressure in the order of 1,000 mmHg, providing a tenfold safety range against acute rupture. Peak force development of the vascular smooth muscle cells (SMCs) occurs at typically ~90 % of the distended passive diameter at 100 mmHg, and allows constriction against ~200 mmHg. Such properties hold over many orders of arterial branching, indicating that mechanisms exist for their maintenance during development and outgrowth of the arteries. These mechanisms of structural control are crucial not only for development, but also for vascular inward remodeling in response to a wide variety of physiological and pathological stimuli [34]. Yet, they are only partly identified.

The in vitro or in vivo study of SMC cell–matrix and cell–cell interaction during arterial remodeling could identify fundamental mechanisms of tissue organization. Examples of such studies in small arteries address the cell relengthening and reorganization [20, 21] and the activity of transglutaminases [2] in the vascular wall during early inward remodeling. Still, the complex composition and architecture of even the isolated small artery has provided quite a challenge for such approaches. As an example, the passive diameter of arteries at high operating pressures is believed to be dominated by organization of the collagen backbone. Yet, it is not clear whether adventitial or medial collagen is relevant, and which cell type is effectuating the organization (fibroblast, SMC, invading leukocytes). Moreover, it is not clear whether the stiffening in early inward remodeling indeed reflects modification of the collagen backbone. Alternatively, such remodeling could be effectuated by other matrix and cytoskeletal fibers.

An additive strategy is the study of cell–cell and cell matrix interaction in well-defined artificial systems, with specific cells and matrix elements. Such models include the compaction of collagen matrices by SMC. A single SMC is able to locally remodel the collagen fibril organization by microscopic movement of cell protrusions [12, 14, 17, 22, 24, 25, 29]. This process is independent of rapid contractions [10], as evidenced by the lack of effect of myosin light chain kinase and protein tyrosine kinase inhibitors. We previously developed technology for the study of tractional forces exerted by a single SMC on the underlying substratum [33]. Collagen compaction has also been studied at the macroscopic level, where the area of a disc of collagen densely seeded with SMC is monitored over time. However, these studies generally provide little mechanistic insight, and it is not clear whether such remodeling represents a mere summation of single cell behavior, or reflects properties emerging in clusters and monolayers of SMC. Such synergistic effects could be based on physical restriction of protrusion movement or coordinated cooperation between cells that are in contact with each other, thereby providing a much more efficient and powerful way to remodel tissue as compared to single cell compaction.

Here, we study remodeling of collagen matrices by vascular SMC in models of increasing complexity, ranging from single cells to polarized monolayers. We quantitate the dynamics, reversibility and spatial extent of remodeling, and demonstrate a transition from isotropic collagen compaction by single cells to highly anisotropic compaction by pairs of cells and in monolayers, where remodeling occurs predominantly in a direction perpendicular to the cell axis. In addition, considering the crucial role of Transglutaminase 2 in vascular inward remodeling [2, 4], we tested the involvement of this pleiotropic enzyme in the remodeling of these model systems, aiming especially at its cross-linking function.

## Materials and methods

### Transglutaminase 2 KO mice, cell culture and transfection

TG2 KO mice were originally obtained from Prof. G. Melino (Rome, Italy) and bred at our local facility. The mice are on a mixed C57BL6/SVJ background. Cultured small artery SMCs were obtained by the explant method from mesenteric small arteries of mice (WT and TG2 knock-out having a mixed Bl6/SVJ background) [18]. The arteries were cut in small segments (≈1 mm length) and placed on the bottom of a culture flask filled with Leibovitz medium, supplemented with 20 % heat-inactivated FCS and antibiotics (penicillin/streptomycin). After 2–3 weeks of initial growth in L-15 medium with 20 % (*v/v*) heat-inactivated fetal calf serum (HI-FCS; Invitrogen), cells were trypsinized, suspended in L-15 medium with 10 % (*v/v*) HI-FCS, and seeded in 25 cm^2^ plastic culture flasks (seeding density: 3,200 cells/cm^2^). Typically, cells achieved confluence within 3 days and then cell number was constant for 1 week. Cells from passages 3 to 9 were suspended in L-15 without serum and used for experiments.

A SMC line (MOVAS, ATCC CRL-2797) was cultured in Dulbecco’s modified Eagle’s medium (DMEM, Invitrogen) containing 10 % fetal bovine serum (Gibco) and a mix of antibiotic–antimycotic (Gibco).

### Immunofluorescent staining of contractile markers

The phenotype of SMCs under normal culturing conditions was studied by immunofluorescent staining of several contractile markers. MOVAS, explant wild-type or TG2 knock-out cells were trypsinized and reseeded in microscopic culture chambers (BD Falcon 354102, untreated glass). After 24 h, cells were washed with warm PBS and fixated with formaline (20 min on ice). Cells were permeabilized with 0.05 % Triton X-100 and blocked with 3 % BSA/5 % goat serum. Samples were then incubated for 1 h at room temperature with mouse monoclonal antibodies against either α-actin (DAKO MO851, 1:500), calponin (Sigma C2687, 1:1,000) or myosin heavy chain (Abcam Ab-683, 1:400). Subsequently, anti-mouse Cy3 (Brunschwig 115-165-166, 1:300) was used as secondary antibody, and slides were mounted in Vectashield/DAPI (Vector Laboratories H-1500). In addition, rabbit monoclonal anti-mouse smoothelin (gift from Guillaume van Eys, Maastricht University, 1:1,000) followed by anti-rabbit Cy3 (Brunschwig 111-165-144, 1:300) were employed. Cells were then visualized using a Leica confocal microscope (TCS SP2).

### Preparation of collagen matrix

Soluble calf skin collagen was purchased from MP Biomedicals. Collagen was dissolved in acetic acid 0.2 M; collagen solution was neutralized by titrating with NaOH 2 M in the presence of HEPES buffer and then water was added to reach the desired collagen concentration. The collagen concentration used in compaction experiments was 1 g/l. Gels were obtained by polymerization for 1.5 h at 37 °C. After polymerization, the gel was repeatedly washed with either L-15 or DMEM in order to bring the ionic composition, pH and osmolarity of the gel to that of the culture medium. Dimensions of disks or rings of matrix without cells were never observed to change for tested periods of up to around 7 days at 37 °C.

### Microscopic compaction of collagen matrix

Tissue remodeling by a reorganization of the collagen architecture was studied using SMCs that were sparsely seeded on a collagen gel. Experiments were performed in plastic cell culture wells (surface 3.8 cm^2^) coated with a 300 μm thick collagen layer (1 g/l) and filled with L-15 culture medium. The contribution of TG2 to microscopic compaction was established using cells from either WT or TG2 knock-out (KO) cultures. Cells were resuspended in L-15 + HI-FCS (10 %) to inactivate trypsin and washed with L-15 after adhesion to the collagen substrate to achieve a final suspension with <0.5 % (*v/v*) HI-FCS. We aimed to obtain one cell per microscopic field; this resulted in an optimal seeding density of ~2 cells/mm^2^. Seeded cells were allowed 20 min to attach to the collagen matrix. After seeding, wells were incubated at 37 °C in a transparent incubator on the microscope stage. Cells were allowed to compact the gel for 24 h and then cytochalasin D (final concentration 10^−6^ M) was added to disrupt cytoskeleton and assess reversibility of compaction [24, 25, 36]. In additional experiments in DMEM, the effect of the TG active site inhibitor L682777 [5, 28, 32] (Zedira, T101: 5 μmol/L, also known as R283) was tested.

The setup used for microscopic imaging of individual cells has been described elsewhere [33]. Time-lapsed video recordings for each position were manually screened off-line for image quality and cell activity. We have used very low cell densities in these experiments, and have selected cells without any close neighbors. No rigid selection criterion was used for this, but presence of cells just outside the field result in highly asymmetric deformation profiles. Such cases were excluded from the data. Series of images (stacks) having good optical quality and active cells were analyzed using a nested cross-correlation algorithm as previously described [33]. The cross-correlation relied on the ability to recognize the pattern of individual spots in the collagen, which had a fibrous texture, in successive images. We defined initial circles with various initial radii (*r*_*i*0_ = 96, 131, 166, 201, 236, 271, 306 μ for *i* = 1–7), centered on the center of the cell, and followed the fate of these circles (Fig. 2 in the results). This way, we determined the new enclosed area and calculated the area change (Δ*A*_*i*_ = *A*_*i*_ *−* *A*_*i*0_). Average radial matrix displacement (Δ*r*_*i*_ = *r*_*i*_ *−* *r*_*i*0_) corresponding to this area change was then calculated using the formula:

1 $$ \Updelta r_{i} = \sqrt {\frac{{\Updelta A_{i} + \pi \cdot r_{i0}^{2} }}{\pi }} - r_{i0} $$

Negative values here reflect movement of gel towards the cell. Strain between consecutive circles within the gel (*i* and *i* + 1) was calculated based on the initial radii (r_*i*+1,0_, r_*i*0_) and maximum displacements corresponding to each radius (Δ*r*_*i*+1_, Δ*r*_*i*_) using the formula:

2 $$ \varepsilon = \frac{{\Updelta r_{i + 1} - \Updelta r_{i} }}{{r_{i + 1,0} - r_{i0} }} $$

where strain is negative for compaction. Cells that crossed the inner circle at any time point were excluded from the data.

### Macroscopic compaction of collagen matrix

Tissue remodeling during organization of the cellular and intercellular architecture and proliferation towards a monolayer was tested in DMEM, using a circular collagen matrix, allowing subsequent mechanical testing. An in-house fabricated Teflon mold (15 mm diameter) was placed in the center of a six-well plate (35 mm diameter) with ultra-low binding coating. Then 2 ml of collagen solution was added around the mold, the gel was allowed to polymerize and repeatedly washed with medium. Immediately after seeding MOVAS cells, the gel was detached from the edges of the well and the central mold was removed. The contribution of TG2 cross-linking to macroscopic compaction was assessed using the active site inhibitor L682777.

Macroscopic compaction was quantified based on reduction of the outer and inner perimeter of the gel, which were imaged after 6 and 24 h. Anisotropy of compaction was estimated from the changes in perimeters and was expressed as an anisotropy parameter α, with α = 1 indicating isotropy and α > 1 preferential radial compaction. Parameter α was calculated as the relative change in distance between inner and outer contour divided by the relative change in midline circumference.

The underlying cellular processes during global compaction were studied using time-lapsed microscopy [33]. In order to limit gel movement during automated horizontal movement of the microscope stage, the amount of medium was decreased as compared to the macroscopic observations. We defined three distinct phases of 100–140 min during which images were taken at a 3-min interval. In the first period, starting about 2.5 h after cell seeding, SMCs developed a preferential alignment. After 8 h, the initial formation of cellular networks was investigated. One day after cell seeding, cell–matrix interactions of a confluent layer of SMCs were studied. During the latter two phases, compaction was calculated locally in both the radial and circumferential direction. First, an independent observer picked cell pairs that were either aligned parallel or head-to-tail in the first image of a time series. Another observer measured the distance between paired cell centroids at the beginning and end of each time series. Local compaction was then calculated as the change in distance divided by the initial distance.

About 72 h after cell seeding, each gel was mechanically tested in a wire myograph (Danish Myo Technology) [34], comparable to vessel ring techniques. For this, the gel was placed around two clamp screws such that force development was just above threshold. Then, the gel was strained about 35 % over a period of 7 min and several parameters were calculated to characterize the visco-elastic properties of the gels. These were the slope of the linear part of the stress–strain curve during the gradual stretch, and the stretch–relaxation behavior, quantified as the percentage of the peak force that remained in steady state (taken at 15 min) and the time required to drop to 50 % of this steady state force.

### Statistical analyses

Results are given as average ± SEM. One-way ANOVA with Bonferroni correction was used to assess statistical significance, unless otherwise specified. Significance level was set at *P* = 0.05. Statistical analysis was performed using SPSS 16.0 for Windows.

## Results

### Cultured SMCs exhibit a synthetic phenotype

**Fig. 1:**
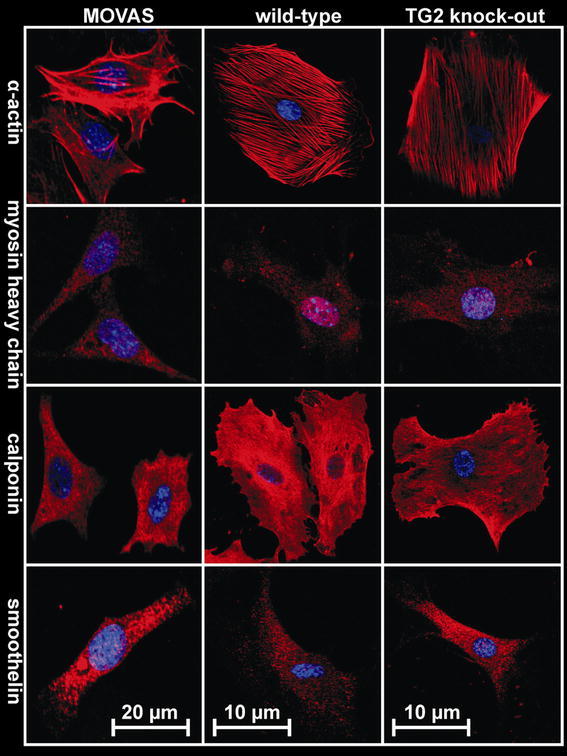
Phenotypical characterization of smooth muscle cells. The MOVAS cell line and explant cells obtained from mesenteric small arteries (WT and KO) were stained for α-actin, myosin heavy chain, calponin and smoothelin, followed by cy-3 secondary antibody (*red*); nuclei are shown in *blue* (color figure online)

We characterized the phenotype of the MOVAS, WT and KO SMC used for seeding onto the collagen matrices (Fig. 1). Explant SMCs were typically more spread compared to MOVAS cells. All cells displayed α-actin immunostaining with evenly distributed intensity along the length of the fibers that is characteristic for synthetic SMCs [38]. All three cell types stained positively for calponin, which was distributed throughout the cytosol with an elevated concentration near the cell periphery. Myosin heavy chain staining was very weak and lacked fiber organization. If present, this signal appeared randomly distributed with a slight preference for cellular protrusions. Smoothelin immunostaining was weak in all cell types as well, and was mainly observed in the more elongated cells in a punctate pattern. Together, all these markers indicate that the vast majority of SMCs used in these experiments exhibited a synthetic phenotype [23, 27, 38].

### A single SMC compacts large areas of collagen matrix: role of TG2

**Fig. 2:**
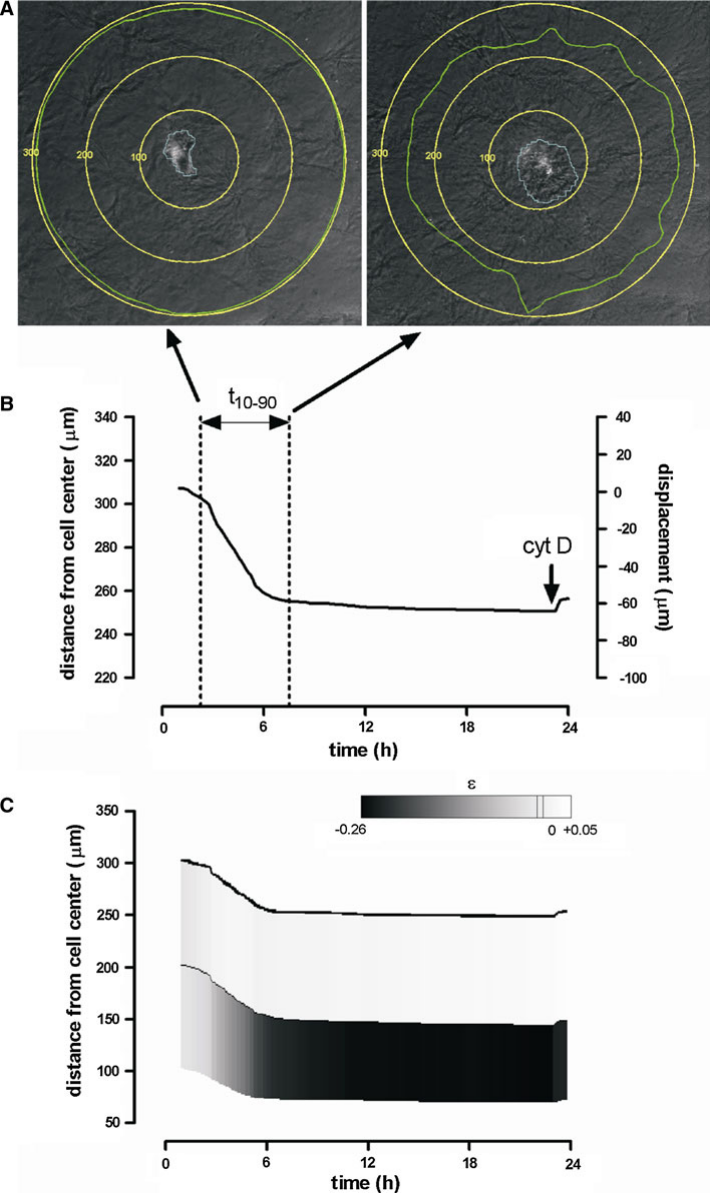
Typical compaction pattern by a single cell. **a** Images acquired after reaching 10 and 90 % of the total compaction. Cell edge is depicted in *blue*, displacement of matrix situated initially on a circle at 300 μm from the center of the cell is followed in time (*green line*). **b** Average displacement in time of a point situated initially at 300 μm from the center of the cell. **c** Mean displacement of matrix originally situated at three distances: 100, 200 and 300 μm (*yellow circles* in **a**). Strain is given by a *gray scale*; *white* depicts expansion and *black* depicts compression (color figure online)

Figure 2a shows a typical example of matrix compaction by a single SMC, imaged at the moment of 10 and 90 % of the maximal compaction. The nested cross-correlation algorithm could be used reliably to calculate the displacement field at a distance larger than 100 μm from the cell center. Matrix displacement was not analyzed closer to the cell due to artifacts resulting from cell locomotion [33]. The largest virtual circle that was tracked (300 μm) displayed a strong, continuous inward displacement over a period of about 8 h, after a short initial lag phase. Cytochalasin D, administered after 24 h, partly reversed gel displacement (2B), indicating a cytoskeletal contribution. The matrix was inwardly displaced up to the tested distance of 300 μm. Between 100 and 200 μm the gel in this example was locally compacted. However, between 200 and 300 μm the matrix expanded locally, as indicated by the positive strain values in Fig. 2c.

**Fig. 3:**
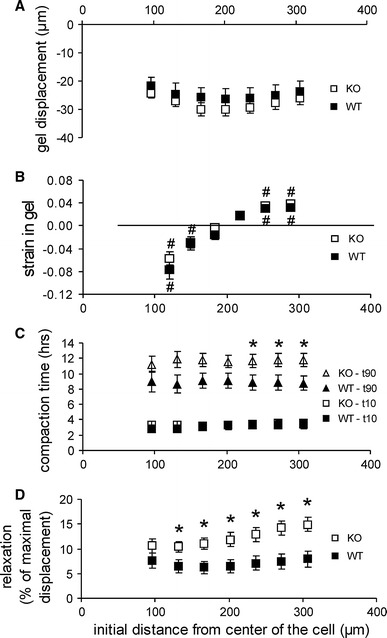
Matrix compaction by individual SMCs from wild-type (WT) and TG2 knock-out (KO) mesenteric arteries. **a** The maximal displacement was not statistically different between WT or KO cells at any measured distance. **b** Both WT and KO SMCs compacted the matrix at a distance up to 200 μm; between 200 and 300 μm, the matrix expanded locally, while this area moved to the cell center as well. **c** The time required to achieve the initial 10 % of the maximal displacement was not statistically different between WT and KO cells, but at distances >200 μm the compaction was significantly faster for wild-type SMCs. **d** Cytochalasin D after 24 h of compaction induced expansion of the matrix due to the loss of cellular contractile forces. The amount of relaxation was significantly lower in WT cells. *Asterisk* different from zero: *P* < 0.05 (**c**, **d**). *Hash symbol* WT versus KO: *P* < 0.05 (**b**)

The degree and speed of matrix remodeling was studied for 36 wild-type versus 52 TG2 knock-out SMCs, divided over five experiments. These data are summarized in Fig. 3. Significant matrix displacement occurred for all tested ranges. We could not track displacement at distances beyond 300 μ from the cell, but an extrapolation of the data in Fig. 3a indicates that displacement is likely to occur over much larger ranges. A significant inward displacement occurred at all tested radii in both WT and KO mice. Maximum radial displacement for WT and KO cells was 30.1 ± 4.2 versus 34.3 ± 2.6 μm occurring at a distance of 200 μm from the cell center (Fig. 3a). Gel displacement was not statistically different for WT and KO cells at any distance. Compaction was maximal closest to the cell for both WT and KO cells, and was significant up to 114, respectively, 149 μm for WT and KO cells (3B). At 184 μm, the gel was merely displaced towards the cell, without a change in radial strain. At distances larger than 254 μm, inward matrix transport coincided with significant expansion (3B). WT and KO cells started matrix remodeling around the same time, as indicated by the almost identical period required to reach 10 % of the maximal displacement. Displacement for WT cells reached 90 % of maximum about 2–3 h faster than for KO cells, which was significant at a distance ≥236 μm. However, while KO cells remodeled matrix for longer times, this was accompanied by a slightly larger maximal displacement (3C). Reversibility of displacement upon administration of cytochalasin D was significant for both WT and KO cells. This reversibility was significantly larger in KO cells for all radii except the smallest one and became larger at increasing distances from the cell center for both WT and KO cells (3D).

**Fig. 4:**
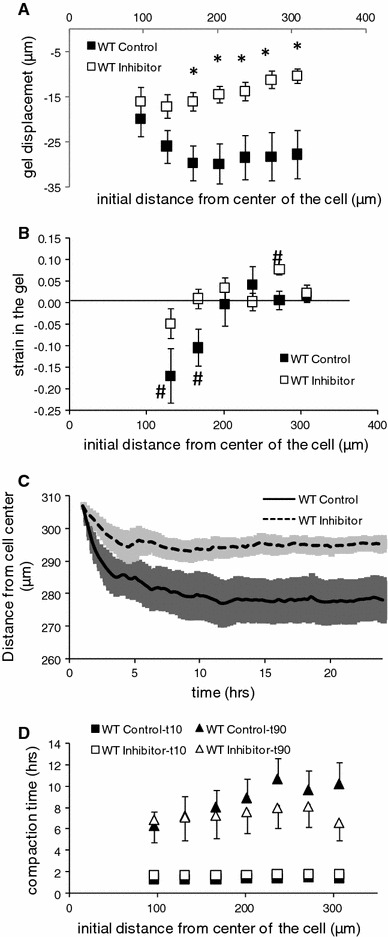
**a** Matrix displacement by WT SMCs in the presence (*n* = 11) versus absence (*n* = 10) of 5 μM of the transglutaminase active site inhibitor L682777. While L682777 had no significant effect on displacement of the innermost circle, compaction between 100 and 200 μm was absent, resulting in statistically significant reduction of displacement that became larger towards the outer rings. **b** Local strain under both conditions. **c** Dynamics of matrix displacement for the outermost ring (307 μm) reveal that this process is slower in the presence of the blocker, and comes to a stand-still after ~5 h, while it continuous for ~12 h in control. **d** Displacement dynamics for the various distances

Transglutaminase 2 KO cells may still produce other TGs [2]. In order to further test the involvement of TGs in matrix displacement and compaction, we studied the effect of the transglutaminase active site inhibitor L682777 on these processes. Figure 4 demonstrates a substantial inhibition. Thus, gel displacement was progressively inhibited at larger distances, the difference becoming significant at 166 μ (4A). This was associated with a full lack of local compaction in the presence of the blocker (Fig. 4b). While we did not determine deformations beyond 300 μ distance, the trend at the higher radii suggests that the range of action of gel deformation is much more limited in the presence of the blocker. Figure 4c depicts displacement dynamics for the outer ring, which appeared somewhat faster in the presence of the inhibitor, although times needed to reach 10 and 90 % of the maximal displacement were not significantly different (4D).

**Fig. 5:**
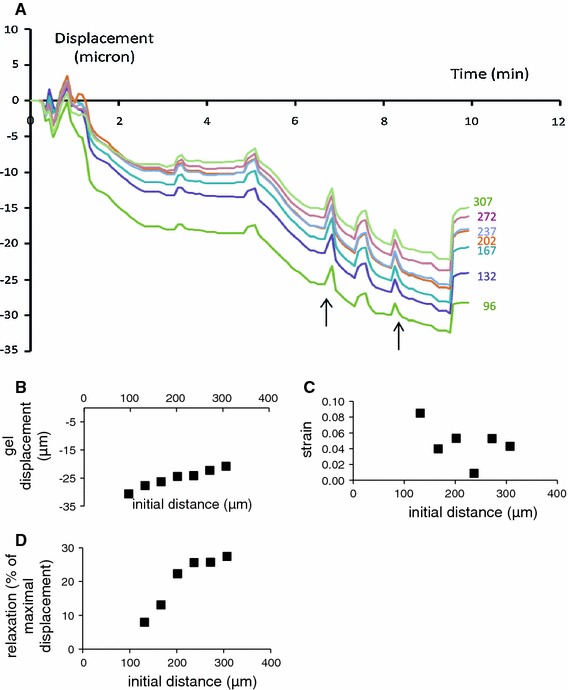
Simulation of matrix attraction by a suction pipette. **a** Displacement at the various distances as a function of time. *Arrow* temporary and final release of vacuum. **b**–**d** Final displacement, strain and relaxation as a function of distance from the pipette center

The above data characterize matrix remodeling as a combination of attraction or ‘hauling up’ exerted by the cell on the surrounding matrix and compaction of the matrix at a distance from the cell. The effects of the active site blocker suggest that the compaction is fully inhibited, while matrix attraction is inhibited to a minor extent. However, gel mechanics are complex, complicating the interpretation of these data. In order to determine the distant consequences, gel attraction by cells was simulated by a suction pipette of 33 μ in outer diameter that was superficially inserted into the gel. Figure 5 depicts gel displacement during application and release of two levels of negative pressure, roughly −0.5 Bar and −1.0 Bar (i.e. full vacuum). Suction resulted in inward displacement of the gel, which was largest for the innermost circle and gradually became less towards the periphery. Actual compaction, as seen for the cells in the absence of the blocker between 100 and 200 μ, was not observed in these experiments. For both vacuum levels, gel displacement slowed down over time towards equilibrium. For −1.0 Bar, displacement above 200 μ progressed towards levels comparable to the cell-based gel contraction, 25–30 μ. As for the cells, only a partial relaxation occurred upon release of the vacuum, increasing from 4 μ (14 %) at 96 μ to 8 μ (30 %) at 167 μ and larger distances.

### Collagen gel compaction by networks of SMCs occurs perpendicular to the SMC long axis

**Fig. 6:**
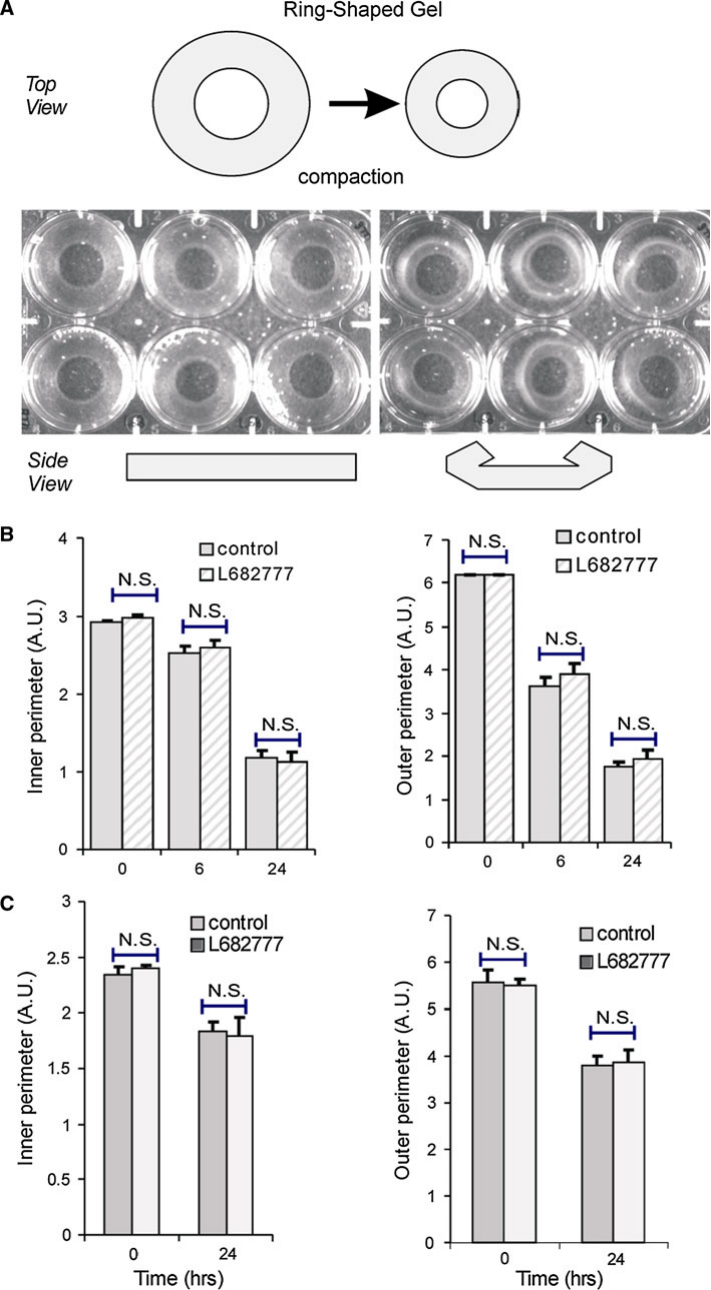
Experimental overview of macroscopic compaction of a collagen matrix. **a** A ring-shaped collagen gel was casted using a Teflon mold in an ultra-low binding multiwell plate; the pictures display the gel at the start and after 6 h of cell-induced compaction, when the gels develop a doughnut shape. **b** Macroscopic compaction, quantified as changes in the inner or outer perimeter of the gel, was not dependent on the TG2 active site inhibitor L682777. *n* = 9 for both groups. **c** Macroscopic compaction in gels that remained flat

**Fig. 7:**
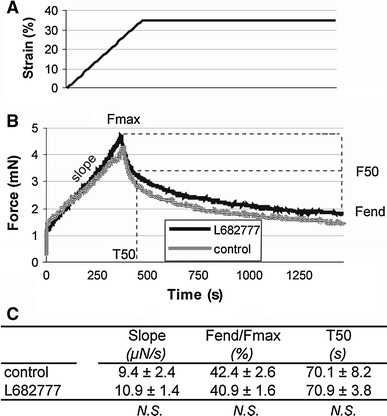
Mechanical characterization of collagen gels (~5 and ~12 mm in inner and outer diameter) after compaction in the presence versus absence of L682777. **a** After 72 h of compaction, gels were uniaxially stretched at a strain rate of 5 % per minute for 7 min, followed by a relaxation phase of 15 min. **b** The visco-elastic gels were characterized by slope, relative decrease in maximal force and the time until half the force decay was reached. **c** L682777 had no statistically significant effect on the visco-elastic properties of the collagen gels. *n* = 6 for both groups

Figure 6a illustrates the compaction of a ring-shaped collagen gel by SMCs. Within 6 h after cell seeding, the outer layer of the gel curled up and folded over the medial layer of the gel. By this time, the outer perimeter had shrunk to 58.5 ± 3.4 % of its initial perimeter. Reduction of the inner perimeter was significantly slower (*P* < 0.01), to 86.5 ± 3.1 % after 6 h. In the next 18 h, the outer perimeter decreased to 28.5 ± 1.3 %, while the inner perimeter shrunk to 40.3 ± 3.3 % of the initial size (inner vs. outer perimeter: *P* < 0.01). After both 6 and 24 h, the decrease in inner and outer gel perimeter was unaffected by the TG2 cross-linking inhibitor (6B, *P* = N.S.). After 24 h, macroscopic compaction had reached steady state. The switch to a doughnut shape may have affected the changes in inner and outer perimeter during compaction. In a next set of experiments, the culture medium level was lowered, causing the gels to remain flat and ring-shaped. Figure 6c shows that also in this case, outer perimeter decreased relatively more than inner perimeter. Also here, L682777 had no effect. After 24 h, the compaction anisotropy parameter α (see “Materials and methods”) was 1.36 ± 0.07 (*n* = 5, *P* < 0.01 vs. unity) in control and 1.23 ± 0.13 in the presence of the blocker (*n* = 5, *P* = N.S. vs. unity, *P* = N.S. vs. control), showing that in control compaction is significantly larger in the radial when compared with the circumferential direction. Mechanical tests in a wire myograph after 72 h of compaction revealed no effect of blocking TG2. Thus, the slope during the straining protocol, the force drop during stress relaxation and the time required for this force drop were not affected by L682777 (Fig. 7, *P* = N.S. for all parameters).

**Fig. 8:**
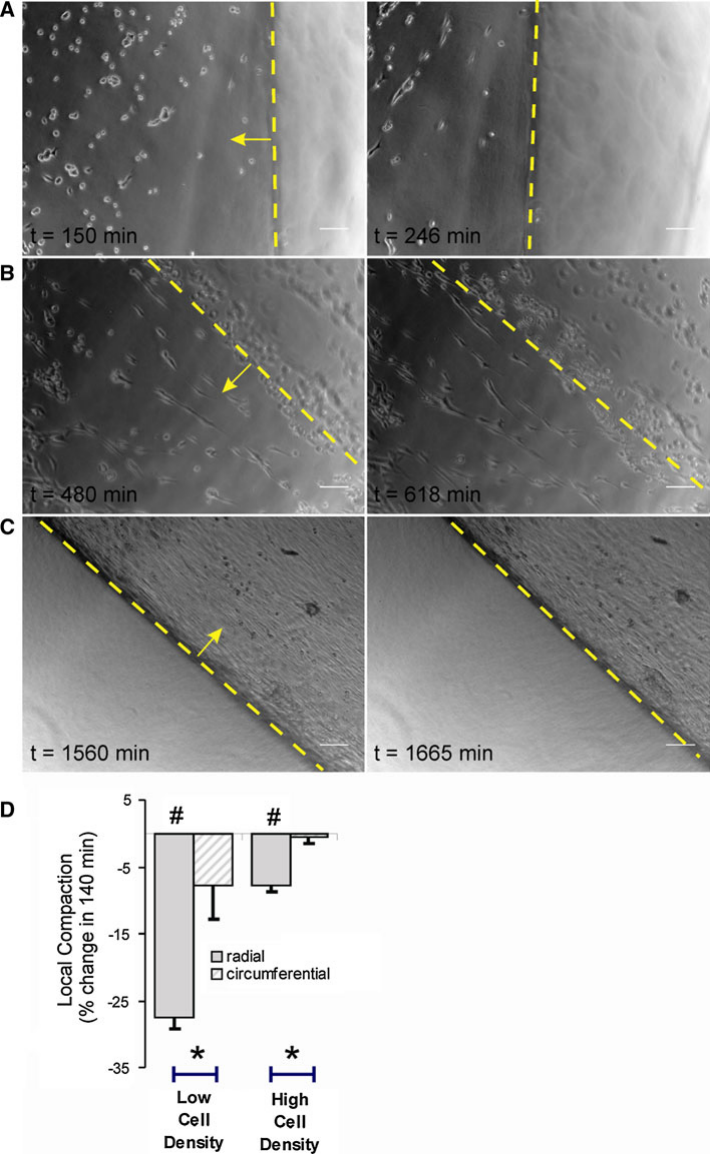
Typical cellular orientation during different phases of matrix remodeling as observed near the inner (**a**, **b**) and outer (**c**) border of the ring-shaped collagen gel model (border indicated by a *dashed yellow line*). **a** About 2.5 h after cell seeding, cells were still round, but already showed a tendency to align along the inner gel boundary; after 4 h, cells have elongated in this direction, while macroscopic compaction was most visible in the perpendicular direction (indicated by a *yellow arrow*). **b** After 8 h, SMC started to develop continuous arrays along the inner boundary, compaction still dominated in the radial direction. **c** Compaction continued after cells had grown to a confluent layer, this was best visible at the outer gel boundary. **d** Quantification of a period of 140 min of compaction starting after 480 (“low cell density”, **b**) and 1,560 min (“high cell density”, **c**). Local compaction at the boundaries occurred especially in the radial direction of the gel, perpendicular to the cellular long axis, and was high at a low cell density. *n* = 19, 13, 26, 27. Analyses were limited to cells that occurred in the same image as the gel boundary. *Scale bar* 100 μm in all panels. *Hash symbol* different from zero: *P* < 0.05; *asterisk* radial versus circumferential: *P* < 0.05 (color figure online)

We next addressed the microscopic changes associated with the macroscopic gel remodeling. Figure 8 shows the cellular orientation during three key phases of gel compaction. About 2.5 h after seeding, cells were still round and started to align in arrays. One hundred minutes later, these cells had partially elongated and their alignment approached the direction of the gel boundary (8A, near inner perimeter). After 8 h, cell density was still low and alignment had proceeded parallel to the gel boundary. About 140 min later, compaction had occurred most notably in the radial direction of the gel, perpendicular to the cells (8B, near inner perimeter). After 26 h, the gel was overgrown by a confluent layer of SMCs, but still exhibited compaction over a period of 100 min (8C, near outer perimeter). At low cell density, such as observed in 8B, compaction was 28 ± 7 and 8 ± 5 % in the radial, respectively, circumferential direction, as judged from the relative movement of pairs of cells. At high cell density, this amounted to 8 ± 1, respectively, 1 ± 1 %. Thus, during the continuous process of cell and matrix organization, local compaction was significantly larger in the radial direction as compared to circumferentially (8D), in accordance with the macroscopic data.

**Fig. 9:**
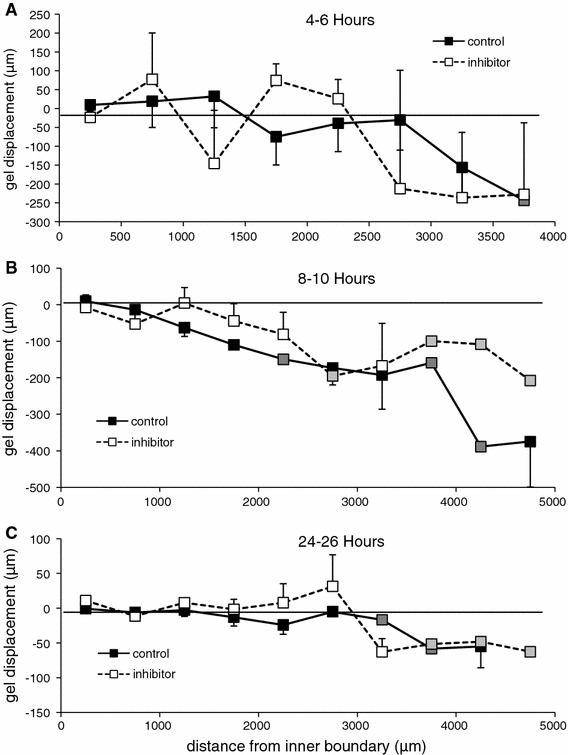
Radial matrix displacement as a function of distance from the inner rim (**a**–**c**). Data were collected by tiling of microscopic images. Attached cells were used as markers, and displacement was determined from the change in perpendicular distance between the cells and inner rim over a 2 h period. Substantial variation was found in local displacement, notably in the presence of L682777. The displacement between 8 and 10 h (**b**) best illustrates that radial compaction (i.e., the slope of the displacement curve) proceeds throughout the gel rather than being a boundary phenomenon. Data were grouped from multiple cells in 500 μm intervals in up to three gels, *error bars* indicate SEM between gels, *grey squares* indicate multiple cells from a single gel. Analysis of circumferential displacement between pairs of cells revealed substantial variation and no differences from zero (data not shown)

The anisotropic compaction may either be a cell property, where elongated cells attract their matrix predominantly in the perpendicular direction, or a matrix property, where radially oriented fibers near the inner and outer perimeter may be more easily displaced. If the latter were the case, one would expect that radial compaction is highest near the inner and outer border. In a next set of experiments, we determined the changes in distance of cells throughout the gel to the inner rim in two hour periods. This analysis was done on a new set of experiments where we tiled multiple microscopic images over time. Figure 9 shows displacement of cells grouped on distance. Local radial compaction is the slope of these curves. These results indicate substantial variability, present both within and between gels. Yet, for none of the time points is a preferential compaction near the inner rim visible. Compaction proceeded the most rapid in the 8–10 h frame, where in the absence of L682777 the compaction (slope) is constant over the whole ring. Results in the presence of the TG blocker indicate more variability, but no significant overall effect.

## Discussion

This work was initiated by the current lack of understanding of cellular and matrix rearrangement in eutrophic inward remodeling of small arteries, which occurs in hypertension, under low flow, or in the continuous presence of a range of vasoconstrictors [34]. We envisioned that a separation of the relevant processes (e.g. contraction, locomotion, compaction) in space and time would allow a more detailed observation and generate hypotheses for further testing at the intact vessel level. We appreciate that the current matrix experiments do not resemble vascular remodeling in several respects, but we do identify some key processes of possible relevance. These include the very large range of action of remodeling by single cells, the highly anisotropic nature of matrix remodeling, and the involvement of transglutaminases. In addition, the current work is of relevance for vascular tissue engineering.

### Matrix remodeling by individual SMC

The concentric matrix remodeling by single cells disclose two simultaneously occurring remodeling processes: matrix attraction and matrix compaction at a distance from the cells. Matrix attraction by single cells was a continuous process, occurring at a relatively constant speed after an initial lag phase of around 2–3 h, resulting in concentric inward remodeling of the matrix. The continuous and large deformation cannot be established by simple contraction of an attached cell, but rather reflects continuous reorganization of matrix fibrils in the area immediately surrounding the cell by dynamic movements of cell protrusions. The consequences of such matrix attraction extended over at least 300 μm and probably much larger distances. It leaves little doubt that this distance is dictated by the density and length distribution of the collagen fibers and the frictional forces associated with their movement. Indeed, a simulation of this process by aspiring matrix through a pipette revealed essentially the same matrix deformation. Displacement induced by the cells leveled off after ~10 h. This could be due to the counterbalancing of cell-derived traction by the elasticity of the matrix [30], possibly in combination with inhibitory effects of the increased local collagen density on cellular dynamics.

If only the dynamic cell protrusions were involved in matrix displacement, one would expect only positive radial strain over the full matrix, such that displacement becomes gradually less towards the gel periphery. This was indeed found in the suction pipette experiments. However, strain was clearly negative up to 200 μm distance from the cell center (Fig. 3), while we excluded cells that had protrusions outside the 100 μ circle. While we cannot fully exclude that we have overlooked these, a direct interaction of the cells in the ring between 100 and 200 μ seems unlikely. This distant compaction can therefore not be effectuated by direct cell–matrix interaction or membrane-bound enzymes, pointing at a role for secreted enzymes. Below, we will discuss the role of transglutaminases in this process.

Recoil of the matrix upon cytoskeletal disruption was limited. It has been shown that collagen gels can be permanently compacted in the absence of cells in a purely mechanical fashion by means of externally applied forces [13, 15]. If this deformation is large enough, it will induce structural anisotropy by aligning collagen fibers, which proves to be irreversible as a result of non-covalent chemical interactions [13, 15]. Likewise, the low reversibility in the current experiments might relate to such permanent compaction by the cells, independent of covalent cross-linking. The limited reversal in the pipette experiment, although it was somewhat larger than around the cells, is in agreement with this view. These issues require future work that includes mechanically loaded gels and better quantitation of matrix visco-elastic properties.

### Matrix remodeling by clusters and monolayers of SMC

The matrix remodeling by single cells predicts a potential for coordinated remodeling by clusters of SMC. The collagen ring experiments indeed revealed such coordinated remodeling at various stages between seeding and final remodeling by monolayers. Shortly after seeding, cells aligned in arrays, without making physical contact (Fig. 8a–b). These arrays were aligned circumferentially and were most notable near the gel perimeter, which can be regarded as a free surface [6]. It was previously shown that parallel to a free surface, cells interact elastically to form strings [6, 11]. These aligned cells started matrix remodeling as seen for the single cells, but also became gradually elongated. This cell alignment and polarization could be due either to preferential distribution along collagen fibers or result from sensing the traction stresses exerted by neighboring cells [13, 26]. Of relevance for both cases, cells were previously shown to strengthen focal adhesions and cytoskeleton organization in the direction of the largest effective stiffness [6, 7]. Cell alignment is believed to be an important step, since gel compaction was previously shown to be a cooperative effect resulting from mechanical interaction between cells [13]. Indeed, local and global compactions are known to depend non-linearly on cell number [19, 31]. This synergistic effect may arise from the fact that contractile activity along a particular direction renders the matrix stiffer, thus prompting neighboring cells to further pull along it [6].

An unexpected finding in the current study was that reduction of the inner circumference (resembling inward remodeling of arteries) was relatively limited when compared with the reduction of outer diameter and compaction of the matrix. This was the case both in the first series of experiments, where the rings transformed into doughnut shapes, and the later ones where the level of incubation medium was reduced and the rings maintained their shape. Since these matrices exudate water during compaction, area and volume are not constant. Therefore, inner and outer circumferences can change independently as far as geometric reasons are concerned. A homogeneous and isotropic compaction would lead to equal relative reductions of outer and inner circumference and ring thickness. A pure radial compaction force would reduce outer circumference and diameter, while maintaining or even increasing the inner diameter and circumference. The actual deformations here depend on elastic properties and should be considered in three dimensions, aspects that are beyond the scope of this study. The current macroscopic results indicate that the preference for radial compaction is relative, not absolute.

The anisotropic compaction could be a property of the matrix as well as the cells. Since the cells aligned circumferentially, this discrimination is difficult to make. Concerning the role of the matrix, isotropic forces exerted by individual cells close to a boundary would produce a compaction that is highest in the direction perpendicular to the free surface, where stiffness is low [6, 13]. Indeed, in selected cells in images that included the inner or outer rim (Fig. 8), radial compaction was several fold higher than circumferential. Concerning the role of the cells, we searched for pairs of cells that had different orientation, but could not find well-defined cases that remained visible and were not surrounded by other cells. In an alternative approach we considered that, if strong compaction would be a gel boundary effect, it should be largest near the inner and outer rim. The data in Fig. 9a–c make clear that this is not the case. Since the gels were freely floating and measurements needed to be done over multiple, tiled images, it was difficult to get more extensive data over longer times here, and future work should solve some of these practical issues.

Radial compaction continued even after a monolayer had developed. We could not reliably image the 3D structure in these areas, but possibly part of this compaction reflects transition of the cells towards a multi-layered 3D structure. Alternatively, apoptosis may have occurred. Future work ought to address these possibilities and should consider the gel deformations in 3D.

The cell densities in these ring experiments were much higher than in the single cell analysis. This precluded us from making the differentiation between matrix attraction and distant compaction that was done for the single cells. Yet, the anisotropy of the process seems to be at variance with an enzymatic process occurring at a distance from the cells. Altogether, while many issues remain to be solved in these ring-shaped matrices, we believe that the current data make a fair case for anisotropic matrix attraction.

### Role of transglutaminases

The possible role of TGs in matrix remodeling was studied using both SMC from TG 2 KO mice and a selective TG active site inhibitor, L682777. In the single cell experiments, both collagen matrix attraction and distant compaction were still present in the KO cells, with some quantitative differences in dynamics. Larger matrix relaxation upon addition of cytochalasin was found in the KO cells. This difference became increasingly clear at larger distances from the cell. Relaxation in the KO cells approached that found in the cell-free suction pipette experiment. This provides some indication for a differential effect of released TGs from both cell types. The effects of the TG active site inhibitor L682777 were clearer, as it completely blocked distant compaction, with minimal effect on matrix attraction. These results strongly point towards a role for transglutaminases in specifically distant compaction. We previously showed that TG2 KO mice have a mild phenotype, where vascular inward remodeling to flow reduction still occurs, albeit at a slower pace, and is associated with activity of factor XIII, one of the other TGs [3], and such compensation might also have occurred here.

Simultaneously, the same concentration of L682777 had no effect at all on the remodeling or biomechanics of the collagen rings. Possibly, due to the higher seeding density, the matrix attraction processes simply overwhelm any distant effect of released TGs in this model. This would plea against either the role of released TGs in tissue remodeling or the physiological relevance of the current model. As concerns released TGs, our microscopic system used for time-lapse imaging of these thick matrices was not suited for fluorescence imaging. Therefore, we did not determine presence of fluorescent TG constructs or incorporation of fluorescent TG pseudo-substrates. However, using TG2-eGFP constructs, we have recently demonstrated that TG2 can be released from cells in microvesicles. Such microvesicles were retrieved at distant locations on collagen and fibronectin coatings [35]. Further research should quantify such distributions in the thick, compacting matrices.

### Implications for small artery inward remodeling

There are obviously clear differences between the current experiments and the inward remodeling of blood vessels. The currently used SMCs had a distinct synthetic and proliferative phenotype at the moment of seeding onto the matrices. We did not monitor phenotypic changes during matrix remodeling, but the continuous proliferation towards a monolayer indicates that the cells remained synthetic during most of the remodeling process. SMCs in blood vessels are in a differentiated, contractile phenotype [9]. However, when matrix reorganization and synthesis is required, SMCs undergo a transient phenotypic modulation to a synthetic phenotype [27, 38]. This dedifferentiation was also observed in mesenteric arteries while they underwent inward remodeling [8, 37], although the capacity for vasoconstriction seems largely unaffected here [16]. The current matrix stiffness was much lower than that of blood vessels, and the current model was essentially two-dimensional. Despite these differences, some possible implications emerge that may warrant future studies.

First, the anisotropic compaction would allow SMCs to organize into dense circular bundles with very little collagen between the SMCs, and maintain this organization with a relatively low tendency for inward remodeling. Compacting forces along the vessel length, also acting perpendicular to the SMCs, would provide a mechanism for the well-known axial stretched state of in vivo blood vessels.

Second, the current models were mechanically unloaded during matrix remodeling. One might therefore question the relevance for vascular remodeling under physiological pressure. Yet, vascular matrix and SMCs are generally considered to be organized in a parallel fashion, such that total wall tension is the sum of an active and passive component. During vasoconstriction, SMCs carry the tension and effectively unload the matrix. Under this condition, dedifferentiation of only a few SMC to a synthetic phenotype would allow inward remodeling, based on the long ranging effect of individual cells. Moreover, shape of such cells was shown to deviate from the spindle shape at low pitch of contractile SMC in small vessels [1]. Considering that matrix remodeling occurs perpendicular to the cell axis, dedifferentiation of a few cells would introduce a circumferential compaction component, which in the presence of tone of the remaining SMC would act on a relatively unloaded matrix. This is speculative, but it would explain the relation seen between vasoconstriction and inward remodeling.

In summary, we studied collagen reorganization by vascular SMCs. Remodeling by individual SMCs was isotropic and characterized by both local attraction and distant compaction. In clusters and monolayers, cells elongated and compacted ring-shaped collagen scaffolds in primarily the radial direction. Transglutaminases were involved in the distant compaction of individual cells. However, such involvement did not influence the remodeling of collagen rings by SMC. The current approach identifies possible mechanisms for vascular organization and remodeling.
